# Spontaneous Co-Assembly of Cellulose Nanocrystals and TiO_2_ Nanorods Followed by Calcination to Form Cholesteric Inorganic Nanostructures

**DOI:** 10.1021/acs.langmuir.3c00981

**Published:** 2023-06-19

**Authors:** Wenshi Zhang, Xinquan Cheng, Shaw H. Chen, Mitchell Anthamatten

**Affiliations:** Department of Chemical Engineering, Advanced Materials for Photonics and Lasers, University of Rochester, Rochester, New York 14627-0166, United States

## Abstract

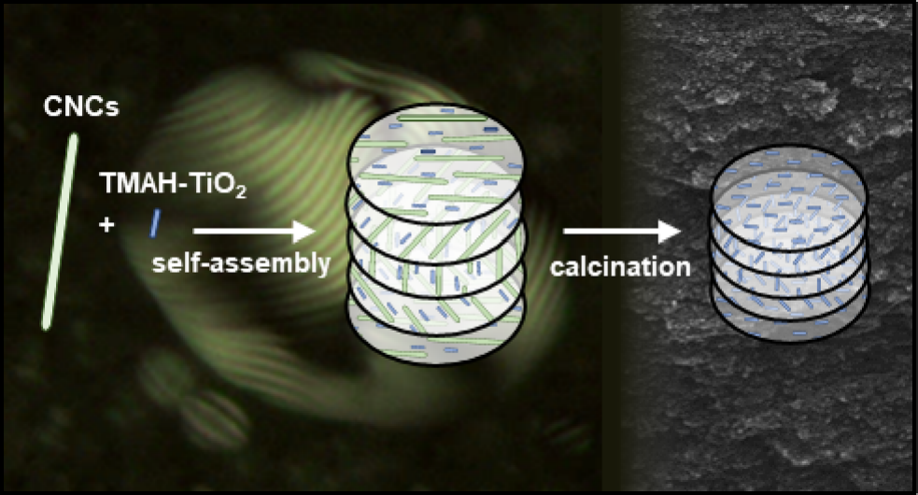

Chiral nanomaterials possess unique electronic, magnetic, and optical properties that are relevant to a wide range of applications including photocatalysis, chiral photonics, and biosensing. A simple, bottom-up method to create chiral, inorganic structures is introduced that involves the co-assembly of TiO_2_ nanorods with cellulose nanocrystals (CNCs) in water. To guide experimental efforts, a phase diagram was constructed to describe how phase behavior depends on the CNCs/TiO_2_/H_2_O composition. A lyotropic cholesteric mesophase was observed to extend over a wide composition range as high as 50 wt % TiO_2_ nanorods, far exceeding other examples of inorganic nanorods/CNCs co-assembly. Such a high loading enables the fabrication of inorganic, free-standing chiral films through removal of water and calcination. Distinct from the traditional templating method using CNCs, this new approach separates sol–gel synthesis from particle self-assembly using low-cost nanorods.

## Introduction

Interest in chiral inorganic nanomaterials has grown due to their unique properties including enhanced chiroptical response, biocompatibility, and superior catalytic activity that open potential applications in chiral photonics, biosensing, chiral separation, and chiral catalysis.^1−4^ Various chiral nanoparticles and chiral assemblies have been reported with optical activities as quantified by their dissymmetry *g*-factors ranging from 10^–5^ to 1.^5−8^ Current methods to prepare chiral nanomaterials generally involve chirality transfer from surface ligands, external field-induced assembly, and template-mediated assembly.^9^ Chiral external fields, such as circularly polarized light or chiral magnetic fields, can impart chirality into nanoscale assemblies.^10−12^ Furthermore, inorganic nanoparticles can be processed into chiral shapes that self-assemble into chiral *supraparticle* structures^8^ analogous to molecular self-assembly forming chiral, supramolecular structures. Nevertheless, fabrication of chiral nanomaterials remains challenging because of (i) the high cost associated with synthesizing chiral nanoparticles, (ii) the precise conditions required for particle synthesis and assembly, and (iii) a limited selection of field-responsive materials.

Chiral templating is a straightforward method for the fabrication of large-scale chiral nanomaterials. Cellulose nanocrystals (CNCs) are naturally abundant and have been utilized as a chiral template owing to their ability to spontaneously form cholesteric superstructures.^13^ Traditional sol–gel hard templating involves infiltration of inorganic precursors into a chiral scaffold followed by sol–gel chemistry to form an inorganic solid and, last, removal of the scaffold. Hard templating can successfully transfer chiral structures into inorganic materials; however, this method requires the fabrication of a mesoporous silica, followed by multiple cycles of impregnation, drying, and template removal to obtain the final material.^14,15^ Soft, chiral templating, on the other hand, involves self-assembly of the chiral template from building blocks, such as small-molecule liquid crystals or chiral nanomaterials, in the presence of precursors. Attempts to directly assemble CNCs as a soft template in the presence of metal oxide precursors are limited to certain types of precursors because of moisture sensitivity, leading to rapid hydrolysis and sol–gel condensation that disrupt assembly.^16,17^ Additionally, the formation of a crystalline product may damage the templated, chiral structure.^16,17^ Thus, there is a need to develop a simple, one-pot assembly method that directly results in chiral, inorganic nanomaterials. We are motivated by the recent demonstration of co-assembly between CNCs and inorganic nanorods to form chiral composite films.^18−21^ However, this bottom-up approach is limited to relatively low loadings (<10 wt %) of inorganic nanorods, and the CNC template was not removed to achieve all-inorganic, chiral structures.

**Figure 1:**
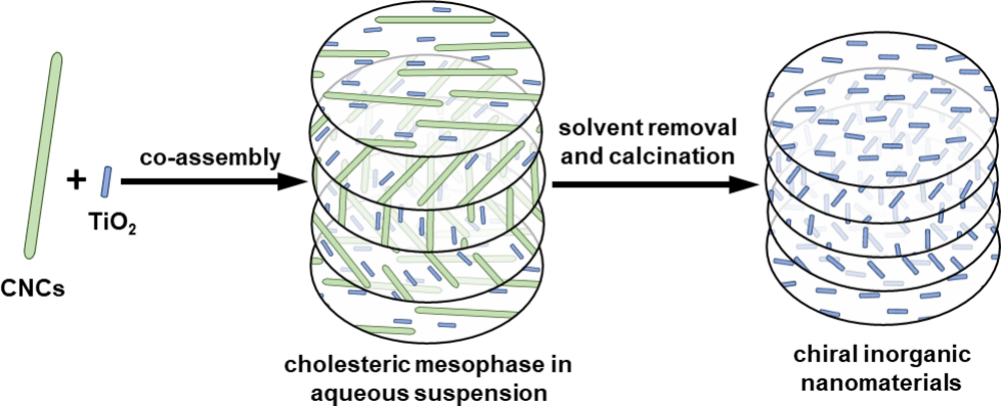
Schematic diagram illustrating co-assembly of CNCs and TiO_2_ nanorods to form a cholesteric mesophase followed by solvent removal and subsequent calcination to remove organics.

Here, we report a bottom-up method to synthesize chiral nanostructures via co-assembly of TiO_2_ nanorods with CNCs that are removed by calcination. A phase diagram describing the mesophase as a function of CNCs/TiO_2_/H_2_O composition is constructed to investigate nanorod co-assembly with CNCs. The resulting phase diagram supports the development of an experimental protocol to create chiral, inorganic superstructures. The results are presented according to our strategy of creating chiral, inorganic superstructures, as illustrated in Figure 1. TiO_2_ nanorods and CNCs are first co-assembled in water at various compositions to form cholesteric, colloidal suspensions, and the system’s co-assembly behavior is captured in a single phase diagram. Next, the ability of TiO_2_ nanorods to couple with a chiral CNC superstructure is evaluated using optical and electron microscopy. Finally, the system’s phase behavior is leveraged to obtain chiral, free-standing films upon removal of water followed by calcination.

## Methods

### Synthesis of TiO_2_ Nanorods

Isopropanol (20 mL) and acetic acid (1 mL) were mixed with TBOT (titanium(IV) *n*-butoxide, 99+%, Thermo Scientific) (10 mL) in a three-neck flask under stirring at 30 °C. Isopropanol (10 mL) and water (1.2 mL) were mixed and then added dropwise to the flask. The mixed solution was further stirred for 1 h before transferring to an autoclave to heat at 150 °C for 20 h. After cooling, the precipitates were washed with isopropanol three times (7500 rpm, 10 min). The obtained TiO_2_ nanorods were dispersed in water around 5 wt %, and then TMAH solution (tetramethylammonium hydroxide, 25% w/w, Alfa Aesar) was added to form a transparent suspension. The weight percentage of TMAH was determined to be around 10 wt % via thermal gravimetric analysis (TA Instruments, Q5000), as shown in Figure S1.

### Construction of the Phase Diagram

A series of TiO_2_/CNCs colloidal suspensions were prepared with varying mass fractions of TiO_2_/overall solid mass ranging from 0 to 1 in increments of 0.1. For each sample preparation, a TMAH-stabilized aqueous suspension of TiO_2_ nanorods was mixed with CNCs (6 wt % in water, Cellulose Lab) to form an aqueous suspension at low overall concentrations in water (∼3 wt %), and the pH was adjusted to about 10 with TMAH. The pH was monitored by narrow range test strips (pH test paper 9.2–10.6, Hydrion). Reverse dialysis according to Liao *et al*.^22^ in polyethylene glycol (PEG) aqueous solution at pH = 10 was performed to concentrate the suspensions up to around 16 wt %. In a typical setup, PEG of a molecular weight of 35,000 g/mol was dissolved in water to form a solution at 20 wt %, and the pH was adjusted to about 10 with TMAH. A CNCs/TiO_2_ suspension was placed in a dialysis bag (SpectraPor Dialysis Membrane, 3.5 kD MWCO) and immersed in the PEG aqueous solution while stirring for a few hours until the desired concentration is reached. The concentration of the suspension was determined gravimetrically, and the suspension was diluted with TMAH aqueous solution at pH = 10 to lower concentrations (2–14 wt %), followed by sonication in an ice bath for 1 h. The gel state of the suspension was determined by placing the sample upside-down, and it was considered to be a gel if it does not flow. The mesophases of the samples were observed under a polarizing optical microscope (POM) in a capillary glass slide (Electron Microscopy Sciences, path × width: 0.4 mm × 8.0 mm), and the observed textures were evaluated to assign mesophases.

### Film Fabrication and Calcination

The suspensions (CNCs/TiO_2_ = 80/20, 6 wt %, 2.0 mL; CNCs/TiO_2_ = 50/50, 8 wt %, 1.5 mL) were sonicated in an ice bath for 1 h before evaporation-induced self-assembly (EISA) under ambient conditions (24 °C, humidity 30–40%). The suspensions were transferred to a 35 mm Petri dish (Polystyrene Cell Culture Dish, 35 mm, Nest Scientific) and the water was evaporated under ambient conditions to obtain free-standing films. The dried, composite films were placed in a furnace (Vulcan 3-550), heated to 400 °C at a ramp rate of 10 °C/min, and held at 400 °C for 4 h to remove CNCs. After calcination, brittle inorganic solid films were obtained, and the film containing CNCs/TiO_2_ at a 50/50 mass ratio appeared more mechanically robust than the 80/20 film.

### Characterization

The dimensions of the TiO_2_ nanorods and CNCs were characterized by bright-field transmission electron microscopy (FEI, Tecnai F20 G2). The suspensions’ zeta potentials were measured using Zetasizer Nano. Powder X-ray diffraction of synthesized rods and the calcined film were conducted using a diffractometer (Rigaku, XtaLAB Synergy-S) with a 2D detector (Rigaku, HyPix-6000HE). The organic fractions of TMAH-stabilized TiO_2_ nanorods, the composite film, and the film after calcination were determined using thermogravimetric analysis (TGA; TA Instruments, Q5000). Prior to each thermogravimetric scan, samples were held at 120 °C under N_2_ for 1 h and then ramped at 20 °C min^–1^ from 120 to 700 °C under air purge. TGA was performed up to 700 °C on a pure CNC sample, and the residual mass was less than 2%. Spectroscopic Mueller matrix ellipsometry (J.A. Woollam, RC2) was performed to collect Mueller matrix data in a transmission mode at normal incidence. The transmission of left-handed and right-handed circularly polarized light was derived from the Mueller matrix spectroscopic data. Scanning electron microscopy (SEM; Zeiss, Auriga) under the InLens mode was utilized to evaluate the morphology of the cross-sectional views of the composite films and calcined inorganic films.

## Results and Discussion

### Nanorod Synthesis and Lyotropic Phase Behavior

TiO_2_ nanorods synthesized following Guang *et al*.^23^ were measured to be 30 ± 5 nm in length and 6 ± 1 nm in diameter (Figure S2a). CNCs (Figure S2b) of 210 ± 50 nm in length and 11 ± 2 nm in diameter were mixed with TMAH-stabilized TiO_2_ nanorods and formed a homogeneous mixture in water. The suspension remained transparent, without sedimentation, over weeks, indicating good colloidal stability. The zeta potential of a 1 wt % suspension containing CNCs/TiO_2_ at 80/20 by mass was measured to be −41 mV, providing further evidence of colloidal stability.^24^

**Figure 2:**
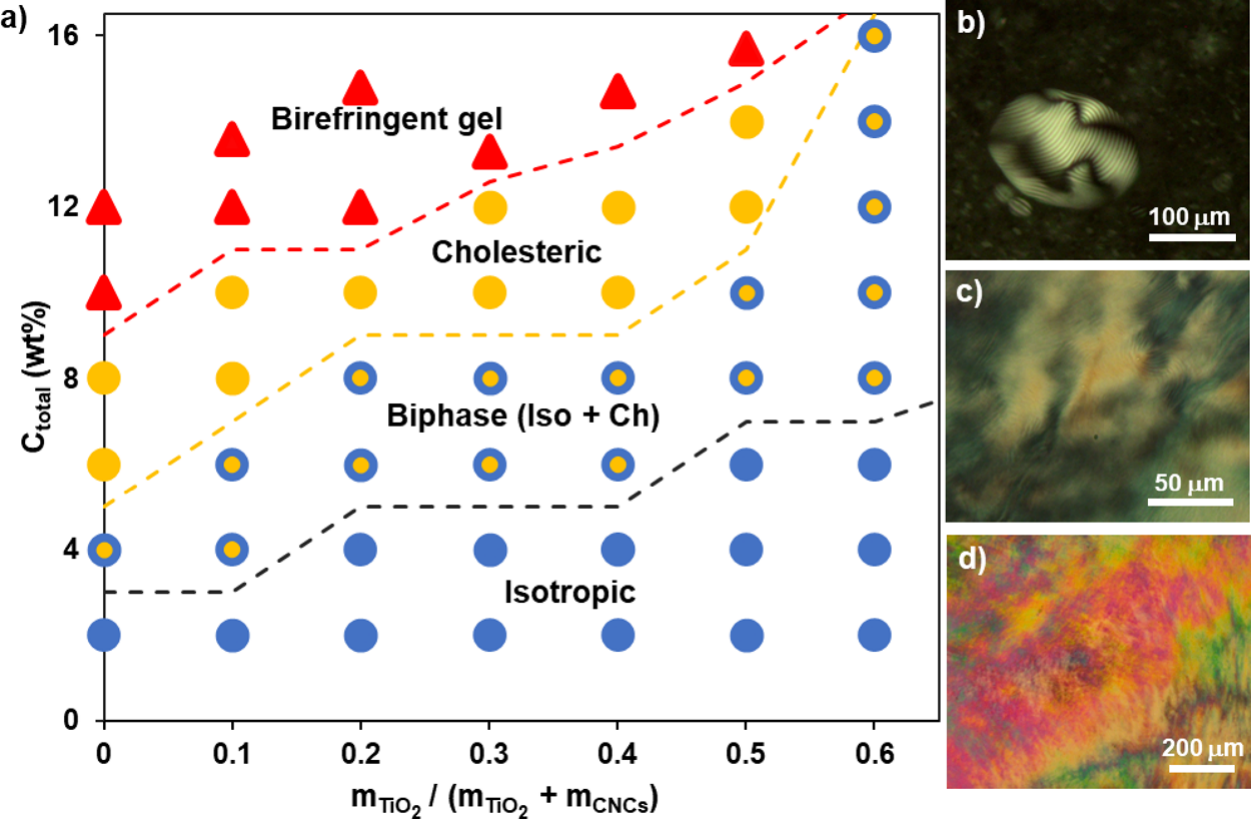
Co-assembly behavior of TMAH-stabilized TiO_2_/CNCs in water: (a) phase diagram of the mixture determined by POM and POM images of (b) the biphasic region containing the isotropic phase and cholesteric mesophase, (c) cholesteric mesophase, and (d) birefringent gel.

The resulting colloidal suspensions containing both TiO_2_ nanorods and CNCs were concentrated to desired concentrations via reverse dialysis in PEG aqueous solution.^22^ The obtained concentrated suspensions did not show observable aggregation. A typical suspension is shown in Figure S3, indicating highly transparent, concentrated suspensions. POM was adopted to determine the mesophases of the colloidal suspensions from the liquid crystalline textures, and the phase diagram of co-assembled CNCs and TiO_2_ is shown in Figure 2, where the *x*-axis of the phase diagram is the weight fraction of TiO_2_ with respect to the total solid (*m*_TiO2_/(*m*_TiO2_ + *m*_CNCs_)) and *y*-axis is the overall solid weight concentration (*C*_total_) in water ((*m*_TiO2_ + *m*_CNCs_)/*m*_suspension_). At low concentrations, an isotropic phase is observed where no light transmits through the POM. As the concentration increases, a biphasic region containing both isotropic and cholesteric mesophases is observed. In a typical POM image (Figure 2b), cholesteric tactoids with bright and dark lines are surrounded by dark, isotropic regions. When the concentration of the suspension further increases, a full cholesteric texture develops with fingerprint textures (Figure 2c). At higher concentrations, the suspension loses its ability to flow, and a birefringent gel is observed (Figure 2d). With increasing weight fraction of TiO_2_ (*x-*axis; Figure 2a), the solids concentration threshold (*y-*axis; Figure 2a) for achieving the lyotropic mesophase increases, as well as the concentration threshold for gelation. These trends are consistent with literature on assembly of hard nanorods of varying aspect ratios and lengths.^25,26^ For example, theory and experimental effort predict and confirm that lyotropic mesophases form at higher concentrations with decreasing aspect ratio of nanorods.^27^ Also, the threshold concentration for gel formation, i.e., particle percolation, is inversely proportional to the nanorods’ aspect ratio.^28^

Our results reveal that a cholesteric mesophase is present over a wide range of compositions, with TiO_2_ loading up to 50 wt %. When the loading of TiO_2_ exceeds 60 wt %, no sign of a cholesteric mesophase is observed under POM. The phase diagram provides guidance for film fabrication with desired optical properties. Whereas in prior reports of co-assembly with CNCs, the loading of nanorods is typically low (up to about 10 wt %),^18−21^ our system enables a higher loading of nanorods, enabling the fabrication of free-standing chiral inorganic films that offer film integrity and mechanical robustness. The ability to achieve a high loading of TiO_2_ nanorods is attributed to the nanorods’ small dimensions and their colloidal stabilization provided by TMAH. The small size of the rod-shaped nanoparticles further provides an opportunity for their intercalation into the CNC’s chiral structure.

**Figure 3:**
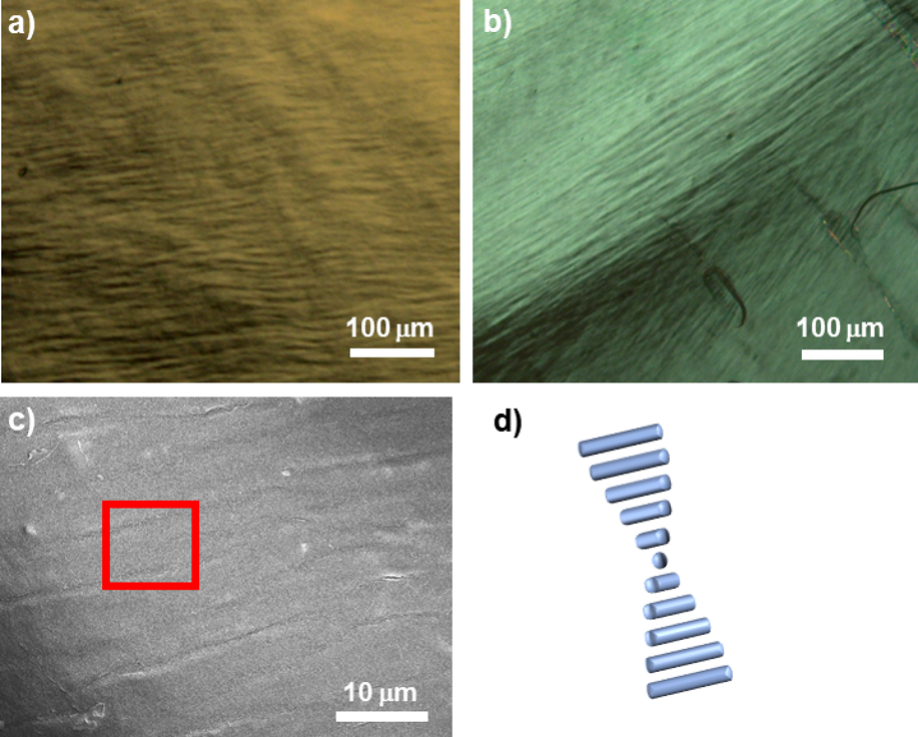
Experimental evidence of coupling between TiO_2_ nanorods and the cholesteric CNC superstructure: POM images of (a) a dried droplet containing 10 wt % solids of TiO_2_/CNCs (20/80 by mass) and (b) the same droplet after calcination; SEM images of (c) the droplet after calcination and (d) a cartoon illustrating the suspected helical arrangement of rods within the red-framed area.

To further verify that TiO_2_ nanorods are coupled to the cholesteric structure of CNCs, a droplet containing 80/20 CNCs/TiO_2_ by mass at 10 wt % in water was dried from the cholesteric mesophase and observed by microscopy, and the results are shown in Figure 3. A typical fingerprint texture—characteristic of a cholesteric structure—is present after solvent evaporation, and a similar texture remains after removal of CNCs by calcination at 400 °C for 4 h, indicating the persistence of the cholesteric mesophase through the thermal treatment process. TGA confirms that calcination successfully removed nearly all of the organic material (Figure S1). Further, there are no crystal structure changes nor observable grain growth according to XRD data (Figure S4) of as-synthesized TiO_2_ nanorods and after calcination. The average pitch length from POM images decreases from 17.0 to 11.5 μm upon calcination, likely due to removal of the CNCs. SEM characterization was also carried out following thermal treatment (Figure 3c,d), and the average pitch length was consistent to that observed under POM. The enlarged area in Figure 3c showed a helical arrangement of TiO_2_ nanorods that could explain the image’s contrast variations.

### Cholesteric Composite Films from EISA

**Figure 4:**
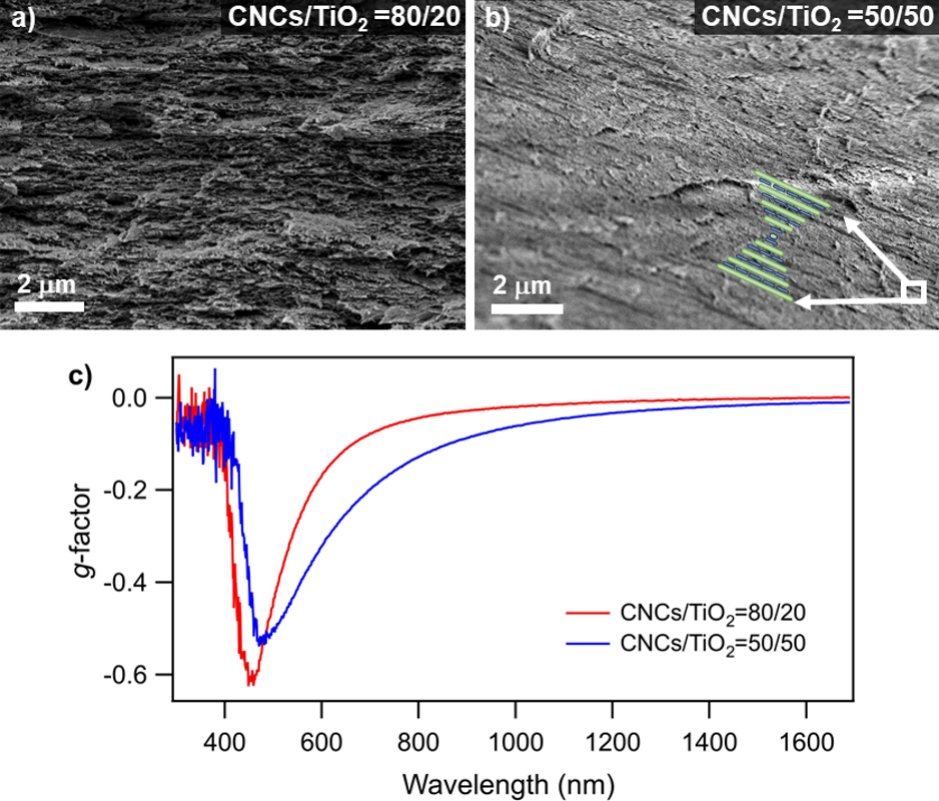
Experimental evidence of chiral superstructures in dried CNCs/TiO_2_ composite films: cross-sectional SEM images of films at (a) 80/20 and (b) 50/50 mass fractions and (c) *g*-factors from spectroscopic ellipsometry.

Free-standing composite films were obtained by EISA of suspensions at selected compositions as guided by the phase diagram in Figure 2. The initial concentrations were chosen in the biphasic regime where cholesteric tactoids could coalesce into larger uniform assembled domains.^29,30^ Two compositions were selected to illustrate the formation of cholesteric, composite films, and dried films of CNCs/TiO_2_ at 80/20 and 50/50 mass ratios were characterized via spectroscopic ellipsometry and SEM. As shown in Figure 4a,b, the cross-sectional view of dried composite films displayed layered structures, resembling cholesteric structures of CNCs. The chiroptical response was quantified by the dissymmetry *g*-factor, defined as *g* = , which describes the preferred transmission of one handedness of the circular polarized light over the other.^31^Figure 4c shows negative *g*-factors for both composite films, consistent with the left-handed cholesteric nature of CNCs that selectively reflect left-handed circularly polarized light. The composite film with CNCs/TiO_2_ at 80/20 exhibits an extremum in a *g*-factor of −0.6 at 450 nm. A slightly lower extremum *g*-factor of −0.5 at around 490 nm was observed for the 50/50 composite film.

Our experimental data in Figure 4 indicate that the suspensions’ composition influences the assembly’s resulting chiroptical response, i.e., the selective reflection of one handedness of circular polarized light. The selective reflection wavelength of the cholesteric structure follows the equation λ_max_ = *n*_avg_*P*, where *n*_avg_ is the average refractive index and *P* is the pitch length. The red-shift of peak’s maximum may be attributed to the higher loading of TiO_2_ as it can increase both *n*_avg_ and *P*. Comparing the 80/20 composite to the 50/50 composite, a higher loading of TiO_2_ nanorods broadens and slightly reduces the intensity of the *g*-factor peak, and this is attributed to dilution of the CNCs and their ability to form chiral structures.

As reported previously, CNCs self-assembly under EISA involves the following steps: (i) phase separation, (ii) tactoid annealing, (iii) gel vitrification, and (iv) film formation.^32^ The self-assembly and related optical properties of the reported cholesteric composite films under EISA could further be optimized by tuning factors such as evaporation rate,^33^ additives,^34^ ion concentrations,^35^ substrate patterning,^36^ and the magnitude of applied electrical or magnetic fields,^37,38^ and these process parameters may be the subject of future study. In addition, vacuum-assisted self-assembly (VASA) is another film fabrication method that can achieve uniformly assembled chiral composite films in much shorter process times^39,40^ and could be applied to the demonstrated CNCs/TiO_2_ composite system. In addition, polydispersity of TiO_2_ nanorods and CNCs may also be a factor in disrupting the assembly.

### Calcination into Chiral, Inorganic, Free-Standing Films

**Figure 5:**
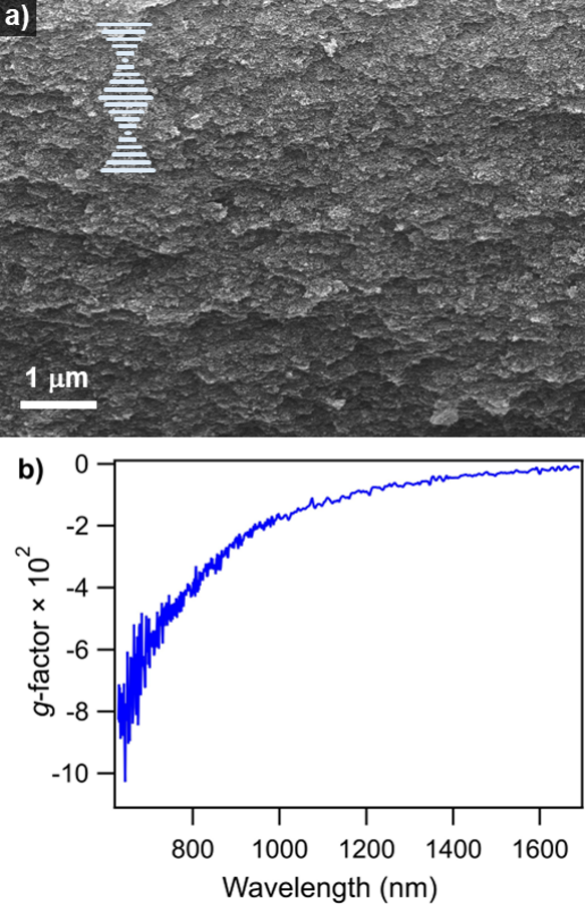
Experimental evidence of chiral superstructures in calcined TiO_2_ films: (a) cross-sectional SEM image and (b) *g*-factor of the inorganic chiral film. The inset in panel (a) depicts a helical structure that is consistent with the layered texture in the SEM image.

The high loading of TiO_2_ nanorods in the composite film offers the opportunity to remove CNCs to form chiral, inorganic films. Thus, composite films with 50/50 CNCs/TiO_2_ from EISA were subject to calcination, and a representative SEM cross-sectional view of a calcined film is shown in Figure 5a. The observed layered structure suggests local nanorod twisting, indicating the transfer of the cholesteric structure from CNCs to TiO_2_. The TiO_2_ nanorods were discernable at high magnifications, as shown in Figure S5. Since the diameter of the nanorods is much smaller than the pitch length, simultaneous imaging of the nanorods and their helical structure is challenging. The chirality is further evidenced by the *g*-factor shown in Figure 5b. An extremum *g*-factor of −0.09 was obtained at 630 nm, indicating a left-handed chiral arrangement. The composite film with CNCs/TiO_2_ of 80/20 was also calcined for comparison. The calcined film is brittle and cracks upon touching with tweezers. However, the layered structure was still observed, as shown in Figure S6a, while the *g*-factor extremum was only −0.03 (Figure S6b). In comparison to our result, Wang *et al*.’s report on the sol–gel templating method involving CNC assembly with TiO_2_ precursors did not result in a cholesteric superstructure after calcination due to the crystallization of TiO_2_ to form anatase.^16^ Our co-assembly of TiO_2_ nanorods with CNCs avoids the sol–gel process, and a chiral TiO_2_ superstructure is realized after removal of the CNC template. Also, the scalable, bottom-up assembly method avoids sol–gel processing toward chiral TiO_2_ films and could be universally applicable to other types of TMAH-stabilized, inorganic nanorods including Fe_3_O_4_ or FePt.^41,42^

There is a clear reduction in chiroptical properties when CNCs are pyrolytically removed. For the 80/20 CNCs/TiO_2_ film, the more significant reduction in the *g*-factor following calcination is attributed to the large volume reduction and collapse of the cholesteric structure. The 50/50 CNCs/TiO_2_ film, on the other hand, has more TiO_2_ nanorods and is more resistive of structural collapse. An improved understanding of changes in structural and optical properties that occur during calcination represents an avenue of future research.

## Conclusions and Outlook

In summary, a simple, bottom-up method toward chiral inorganic nanostructures was achieved by co-assembly of TiO_2_ nanorods with CNCs. The mesophase of the co-assembled suspensions is observed over a wide range of compositions owing to stable suspensions. A lyotropic cholesteric mesophase extends over a wide composition range to the hitherto highest 50 wt % TiO_2_ nanorods. Such a high loading allows for the fabrication of inorganic, free-standing chiral films through water removal and calcination. This scalable, bottom-up assembly method avoids sol–gel processing toward chiral TiO_2_ films and could be universally applicable to other ligand-stabilized, inorganic nanorods. The CNCs/TiO_2_ composite system shows great promise for the fabrication of cholesteric composite films with tunable chiroptical properties, and future study may involve tuning of EISA process parameters or the application of VASA methods.

## Supporting Information Available

Experimental details and additional figures including (i) TGA curves of TiO_2_-TMAH, the CNCs/TiO_2_ composite film, and the calcined free-standing film, (ii) TEM images of TiO_2_ nanorods and CNCs, (iii) transparent suspensions of 8 wt % aqueous suspension containing 50/50 CNCs/TiO_2_, (iv) XRD data of as-synthesized bare TiO_2_ nanorods and the calcined chiral TiO_2_ film, (v) cross-sectional SEM image of the calcined film at high magnifications showing nanorod geometry, and (vi) cross-sectional SEM image and *g*-factor of the calcined inorganic film from the CNCs/TiO_2_ (80/20) composite film (PDF)
