# Supplementary material for: Spontaneous Co-Assembly of Cellulose Nanocrystals and TiO2 Nanorods Followed by Calcination to Form Cholesteric Inorganic Nanostructures
Source: Langmuir. 2023 Jun 19;39(26):9180–5. doi: 10.1021/acs.langmuir.3c00981 (PMC10324389; doi:10.1021/acs.langmuir.3c00981)
Supplement: Supplementary file 1 — la3c00981_si_001.pdf [file la3c00981_si_001.pdf]

Supporting Information

Spontaneous Co-Assembly of Cellulose Nanocrystals and TiO_2 Nanorods Followed by Calcination to Form Cholesteric Inorganic Nanostructures

*Wenshi Zhang, Xinquan Cheng, Shaw H. Chen, and Mitchell Anthamatten**

Department of Chemical Engineering, Advanced Materials for Photonics and Lasers

University of Rochester, Rochester, NY 14627-0166, United States

E-mail: mitchell.anthamatten@rochester.edu

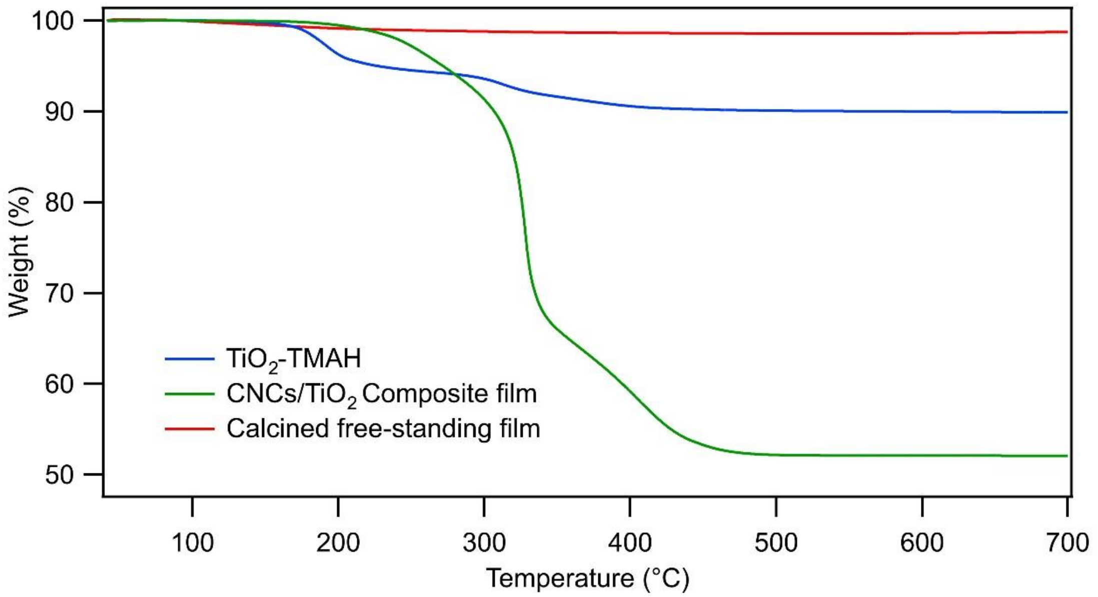

Figure S1. TGA curves of TiO_2-TMAH, CNCs/TiO_2 composite film and calcined free-standing film.

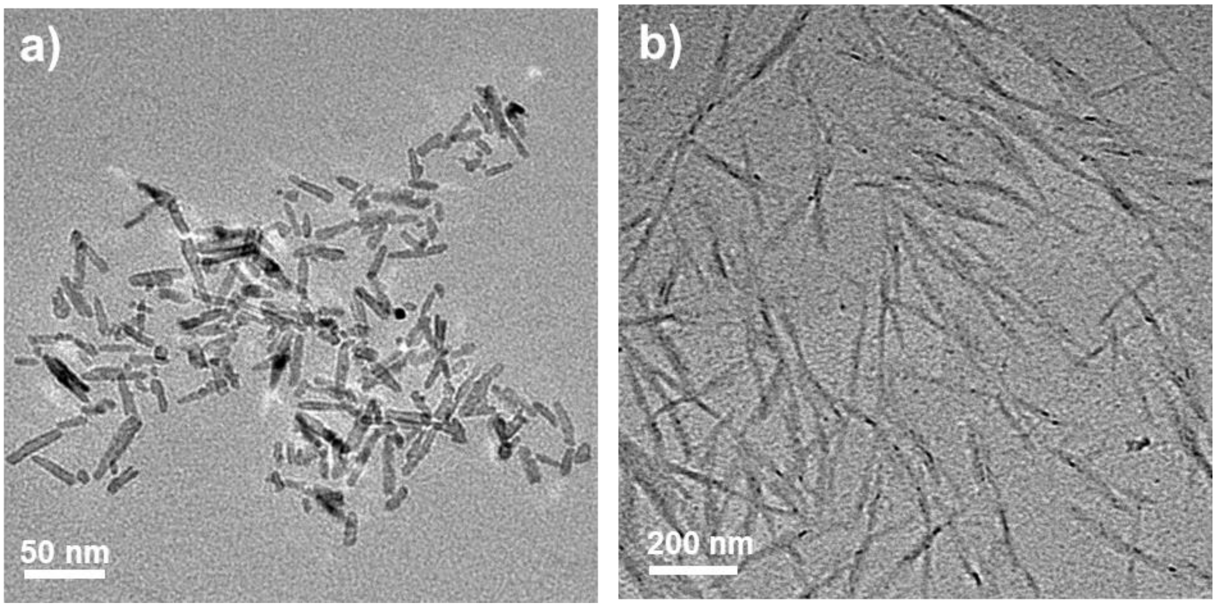

Figure S2. TEM images of (a) TiO_2 nanorods and (b) CNCs.

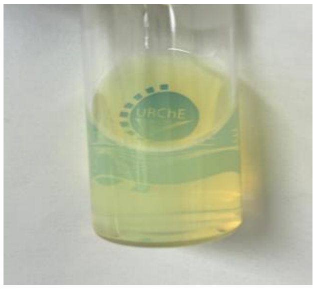

Figure S3. Transparent suspensions of 8 wt% aqueous suspension containing 50/50 CNCs/TiO_2.

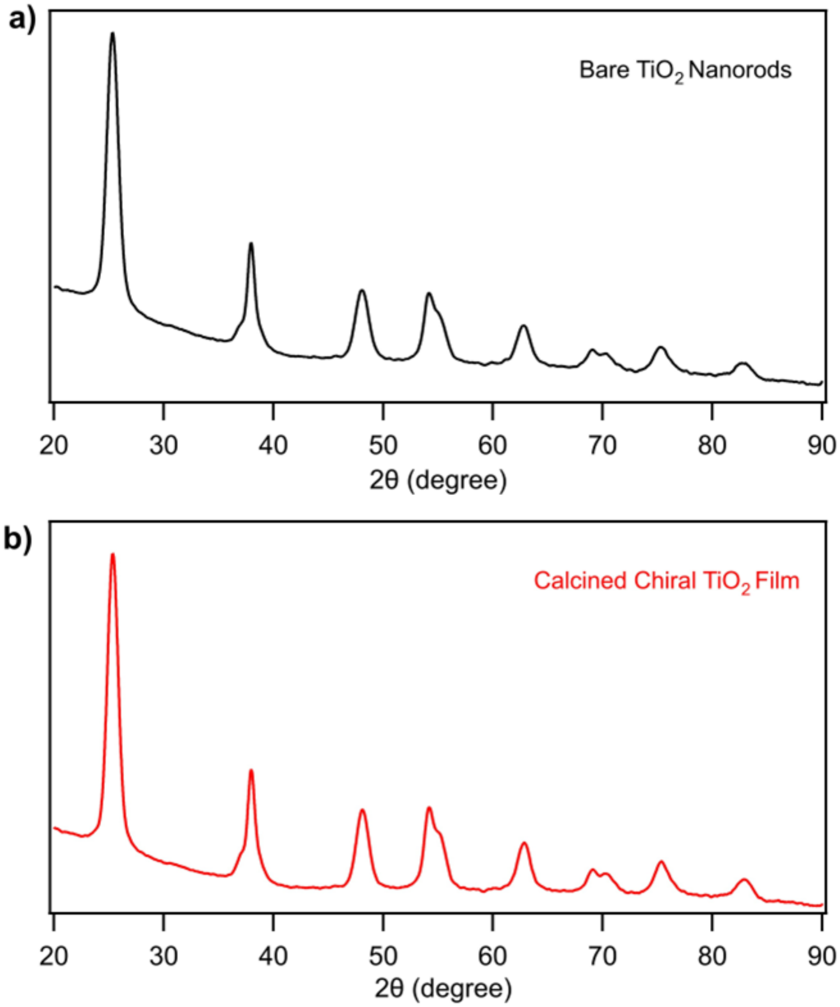

Figure S4. XRD data of (a) as-synthesized bare TiO_2 nanorods and (b) calcined chiral TiO_2 film

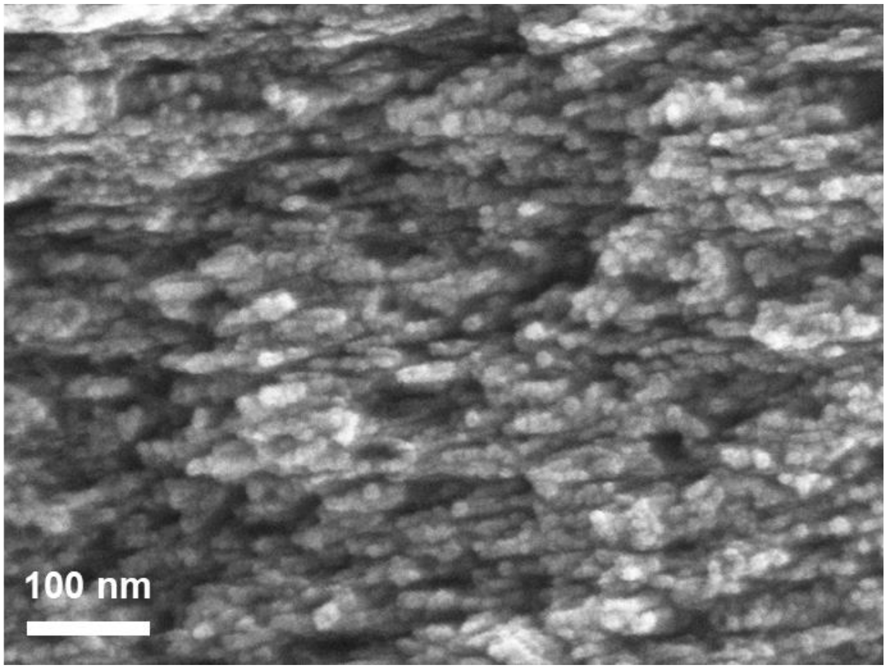

Figure S5. Cross-sectional SEM image of calcined film at high magtification showing nanorod geometry

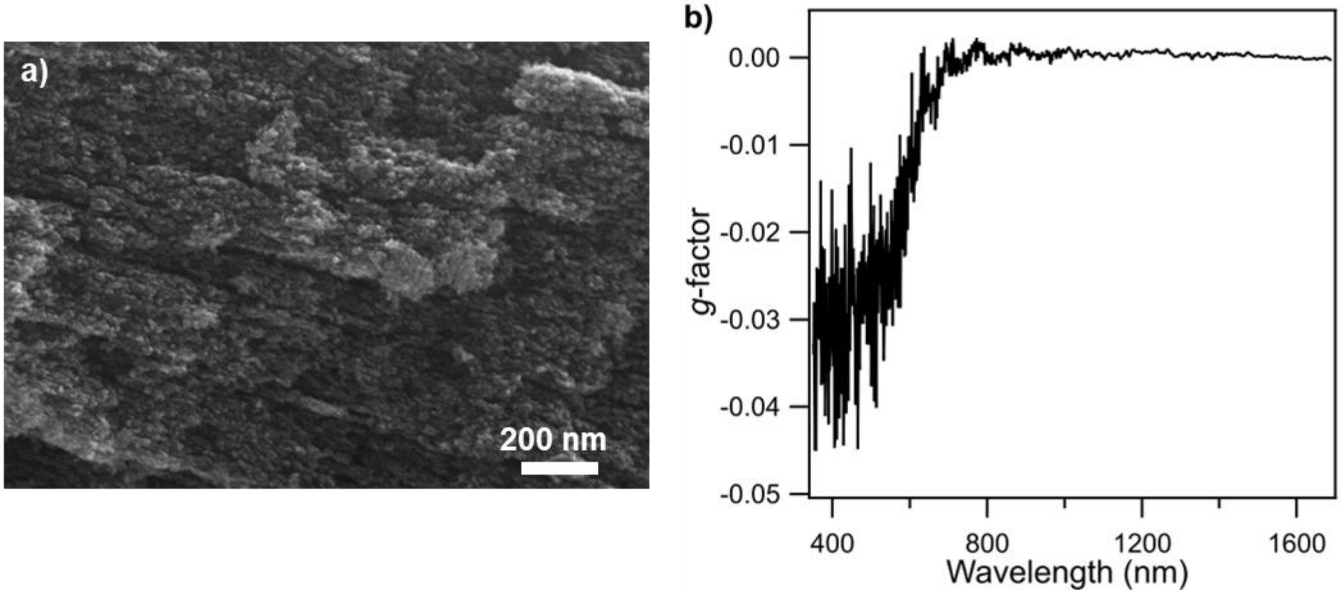

Figure S6. Cross-sectional SEM image (a) and *g*-factor (b) of calcined inorganic film from CNCs/TiO_2 (80/20) composite film
